# Interpretability of radiomics models is improved when using feature group selection strategies for predicting molecular and clinical targets in clear-cell renal cell carcinoma: insights from the TRACERx Renal study

**DOI:** 10.1186/s40644-023-00594-3

**Published:** 2023-08-14

**Authors:** Matthew R. Orton, Evan Hann, Simon J. Doran, Scott T. C. Shepherd, Derfel Ap Dafydd, Charlotte E. Spencer, José I. López, Víctor Albarrán-Artahona, Francesca Comito, Hannah Warren, Joshua Shur, Christina Messiou, James Larkin, Samra Turajlic, on behalf of the TRACERx Renal Consortium, Dow-Mu Koh

**Affiliations:** 1 Artificial Intelligence Imaging Hub, Royal Marsden NHS Foundation Trust, London, UK; 2 Division of Radiotherapy and Imaging, Institute of Cancer Research, London, UK; 3 Cancer Dynamics Laboratory, The Francis Crick Institute, London, UK; 4 Renal and Skin Units, Royal Marsden Hospital NHS Foundation Trust, London, UK; 5 Melanoma and Kidney Cancer Team, Institute of Cancer Research, London, UK; 6 Department of Radiology, Royal Marsden NHS Foundation Trust, London, UK; 7 Biomarkers in Cancer Unit, Biocruces-Bizkaia Health Research Institute, Barakaldo, Spain; 8 Medical Oncology Department, Hospital Clinic de Barcelona, Barcelona, Spain; 9 Medical Oncology, IRCCS Azienda Ospedaliero-Universitaria di Bologna, Bologna, Italy; 10 Department of Experimental, Diagnostic and Specialty Medicine (DIMES), University of Bologna, Bologna, Italy; 11 Urology Centre, Guy’s and St. Thomas’ NHS Foundation Trust, London, SE1 9RT UK; 12 Division of Surgery and Interventional Science, University College London, London, UK

**Keywords:** Radiomics, Radiogenomics, Histology, Interpretable, Machine learning, Feature selection, Group selection, Renal cancer, Nested validation, Molecular subtyping

## Abstract

**Background:**

The aim of this work is to evaluate the performance of radiomics predictions for a range of molecular, genomic and clinical targets in patients with clear cell renal cell carcinoma (ccRCC) and demonstrate the impact of novel feature selection strategies and sub-segmentations on model interpretability.

**Methods:**

Contrast-enhanced CT scans from the first 101 patients recruited to the TRACERx Renal Cancer study (NCT03226886) were used to derive radiomics classification models to predict 20 molecular, histopathology and clinical target variables. Manual 3D segmentation was used in conjunction with automatic sub-segmentation to generate radiomics features from the core, rim, high and low enhancing sub-regions, and the whole tumour. Comparisons were made between two classification model pipelines: a Conventional pipeline reflecting common radiomics practice, and a Proposed pipeline including two novel feature selection steps designed to improve model interpretability. For both pipelines nested cross-validation was used to estimate prediction performance and tune model hyper-parameters, and permutation testing was used to evaluate the statistical significance of the estimated performance measures. Further model robustness assessments were conducted by evaluating model variability across the cross-validation folds.

**Results:**

Classification performance was significant (*p* < 0.05, H_0_:AUROC = 0.5) for 11 of 20 targets using either pipeline and for these targets the AUROCs were within ± 0.05 for the two pipelines, except for one target where the Proposed pipeline performance increased by > 0.1. Five of these targets (necrosis on histology, presence of renal vein invasion, overall histological stage, linear evolutionary subtype and loss of 9p21.3 somatic alteration marker) had AUROC > 0.8. Models derived using the Proposed pipeline contained fewer feature groups than the Conventional pipeline, leading to more straightforward model interpretations without loss of performance. Sub-segmentations lead to improved performance and/or improved interpretability when predicting the presence of sarcomatoid differentiation and tumour stage.

**Conclusions:**

Use of the Proposed pipeline, which includes the novel feature selection methods, leads to more interpretable models without compromising prediction performance.

**Trial registration:**

NCT03226886 (TRACERx Renal)

**Supplementary Information:**

The online version contains supplementary material available at 10.1186/s40644-023-00594-3.

## Background

TRACERx Renal (NCT03226886) [1–3] is an ongoing prospective multi-omics study exploring the genomic and molecular drivers of clear-cell renal cell carcinoma (ccRCC) with a target accrual of 320 patients. The interim results of the first 101 patients provided novel insights into cancer evolution and identified novel prognostic genetic features [3]. Specifically, patient outcome could be independently predicted from weighted genomic instability index (wGII, a measure of chromosomal complexity), and genetic intratumoural heterogeneity (ITH, a reflection of diversity of the genetic landscape of the tumour cells). Directly translating such biomarkers into the clinic is currently not possible since they necessitate cost-prohibitive genomic profiling, the tissue specimens are often compromised by pauci-cellularity, and routine tissue-fixation causes sequencing artefacts. A more intractable problem is that multiple tumour samples are required to accurately capture ITH, which is only possible from post-surgical resection of the tumour. Thus, these biomarkers cannot currently be applied clinically for treatment planning or determining surveillance regimens in patients who do not have surgery. Moreover, for patients who do have surgery, pre-surgical determination of the status of these biomarkers, and of histopathology markers currently evaluated post-surgery (such as the presence of necrosis and of sarcomatoid changes), would be of use in surgical and treatment planning.

Radiomic analysis of pre-surgical contrast-enhanced CT (CE-CT) scans presents an opportunity to non-invasively identify imaging features that can predict prognostic genomic and histopathological metrics, potentially enabling clinical translation of molecular markers via radiological scans. Here we leverage TRACERx Renal to develop accurate radiomic predictors of an array of clinically relevant genomic and histopathological metrics in ccRCC and propose a novel group feature selection method that facilitates radiomic signature interpretability.

There is growing consensus in the radiomics community that whilst obtaining properly validated measures of radiomics model performance is essential, it is also necessary to develop a meaningful interpretation of the predictive model [4–6], and this is true of machine learning (ML) applications beyond radiomics. In a recent review [7], Tomaszewski and Gilles argue that interpreting radiomics signatures in relation to underlying biological processes will be the catalyst for generating robust findings that can impact clinical practice. TRACERx Renal is therefore an ideal study for advancing the field of interpretation in radiomics because the novel molecular metrics of interest have themselves been developed to better understand the underlying biology. In particular, these metrics are influenced by intra-tumour spatial heterogeneity, and whilst this is a natural property of radiomic features, molecular metrics are more commonly derived from a single sampling site.

Feature selection methods are known to improve machine learning model performance and while many studies have assessed the impact of different feature selection methods in the context of radiomic studies [6, 8–10], their effect on radiomic signature interpretability remains unexplored. Radiomics pipelines typically include multiple stages of feature selection that remove features based on properties such as their reproducibility, high correlations with other features, and their relevance when predicting the target of interest [5, 6, 11]. However, standard implementations of these techniques are a priori unbiassed with respect to each feature, meaning that the presence (or absence) of a meaningful interpretation for each feature does not influence the selection procedure.

Determining the interpretability of radiomics features is somewhat subjective, but features derived from the tumour shape (such as tumour volume and surface area) can be easily understood by a lay person, whereas features derived from pixel grey level distributions (such as mean value or entropy) require some specialist knowledge, and features relating to image texture features are not well understood, even by image processing experts.

We hypothesise that using selection procedures that do not account for individual feature interpretability may lead to the discovery of radiomics signatures that are harder to interpret, and so we have developed a novel feature selection strategy designed to nudge the ML pipeline towards discovering more interpretable signatures. Such a biased selection procedure could theoretically compromise model performance, so we compare the prediction performance of models built using different feature selection strategies.

To improve the interpretability of the final signature we employed three approaches: (1) extending the correlation-based feature reduction approach by retaining features with simpler over more complex interpretations; (2) a related supervised feature group selection step that treats the inclusion or exclusion of feature groups from the model as a tuning parameter that can be optimised using cross-validation; (3) use of tumour sub-segmentations to generate augmented radiomic feature sets to relate features in the radiomics signature back to their corresponding sub-segmented regions.

This study aims to:

1. Evaluate the performance of CE-CT radiomics predictions of a range of novel prognostic genetic features and clinical targets in the TRACERx Renal cohort (101 patients), and determine which targets warrant further study in the final cohort (320 patients).
2. Compare the interpretability of radiomics signatures obtained in these data using standard feature selection methods and the proposed feature selection methods.
3. Evaluate the impact of tumour sub-segmentation on radiomics signature interpretability.

## Methods

### Patient cohort

**Fig. 1:**
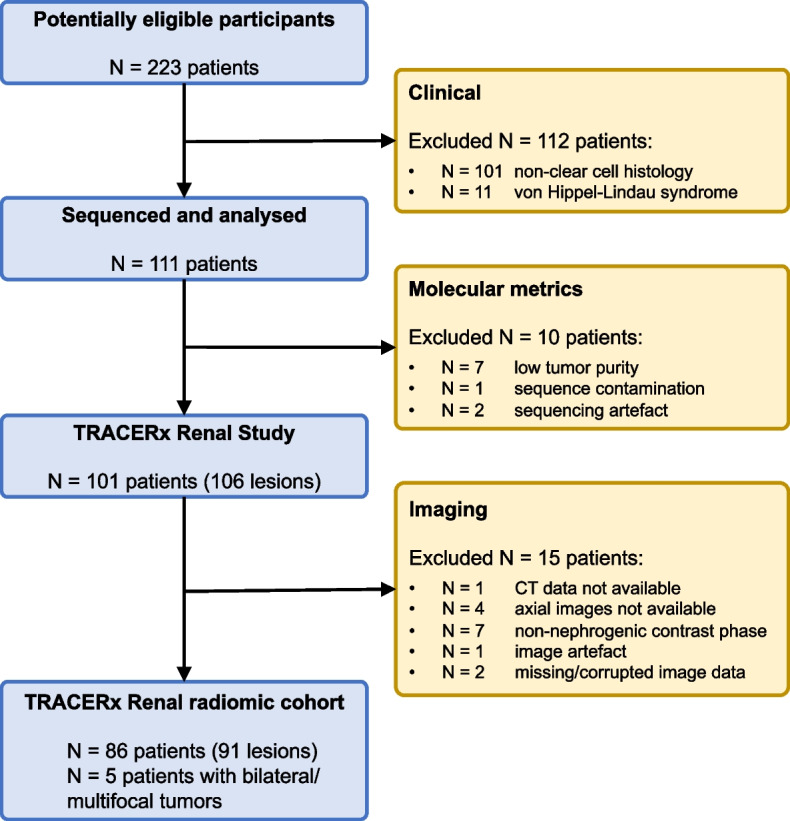
Flow diagram showing patient inclusion and exclusion process

The subjects included in this study are the first 101 consecutive patients recruited to an ongoing prospective study (NCT03226886, TRACERx renal: study started February 2012, estimated completion September 2023, estimated enrolment 320 patients). Ethical approval was granted for this cohort study by the National Health Service Research Ethics Committee (11/LO/1996, UK). Details of patient selection and inclusion/exclusion criteria are outlined in the supplementary information section S1, and 91 lesions were finally available for radiomics analysis, see Fig. 1.

### Molecular and histopathological targets

Table 1 lists 20 classification targets and the number of patients falling into each class for that target. The first six targets in Table 1 are obtained from histopathological examination of the surgical specimen and are currently used in standard of care clinical practice to prognosticate patients after surgery. Overall pathological stage was binarized by grouping into stages 1 or 2 (tumours confined within kidney) vs stages 3 or 4 (tumours not confined within kidney). The 14 molecular targets in Table 1 were derived by multi-regional sampling and next generation sequencing (NGS) of the surgical specimen and include somatic mutations in ccRCC driver genes or somatic copy number alterations (SCNAs), ITH and wGII, and the evolutionary mode of the primary tumour: “linear”, “branched” and “punctuated” evolutionary subtype (EvoST), described in [2, 3]. Further details are in the supplementary information, section S2. Somatic alterations were described as either clonal (present in all regions in the tumour), subclonal (present in a subset) or absent. Prediction of the three EvoSTs was performed as a one vs all classification.

**Table 1:** List of classification targets and abbreviations, and the proportion in the positive class. Targets with ^a^ indicate continuous variables that were binarized by thresholding on the median cohort value – note that the % positive is expected to be 50%, but the presence of repeated values in these targets means this is not exact. Overall stage was binarized as stage 1 or 2 vs stage 3 or 4. The eleven targets in bold font have their predictive models explored in detail in the Results section and Discussion

**Target type**	**Target**	**# negative**	**# positive**	**% positive**
Histopathology	**Necrosis**	41	50	55
	**Sarcomatoid changes**	82	9	10
	**Microvascular invasion**	53	38	42
	**Renal vein invasion**	47	44	48
	**Inferior vena cava invasion**	76	15	16
	**Overall Stage 12 vs 34**	60	31	34
Evolutionary subtype	**Branched**	68	23	25
	**Linear**	82	9	10
	Punctuated	66	25	27
ITH and chromosome complexity	**ITH Index**^**a**^	49	42	46
	**wGII Max**^**a**^	45	46	51
	wGII Median^a^	44	47	52
Somatic events	**Loss 9p21.3**	36	55	60
	Loss 9p21.3 isClonal	73	18	20
	Loss 14q31.1	32	59	65
	Loss 14q31.1 isClonal	68	23	25
	BAP1	72	19	21
	BAP1 isClonal	80	11	12
	PBRM1	41	50	55
	PBRM1 isClonal	54	37	41

### Image preparation and feature extraction

Pre-surgical CE-CT scans were obtained during the nephrographic contrast phase from four different scanner vendors using a range of standard of care imaging protocols detailed in supplementary information Table S1. Scan data were saved in DICOM format prior to being pseudonymised and transferred to a research PACS based on the eXtensible Neuroimaging Archive Toolkit (XNAT) platform [12], which served as the principle repository for image curation and analysis.

**Fig. 2:**
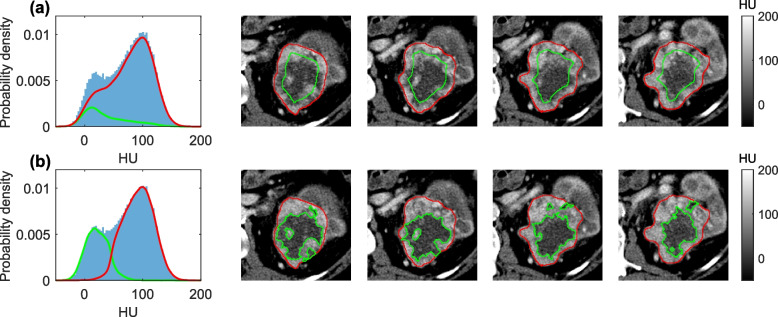
Example tumour sub-segmentations and histograms: **a** rim/core and **b** high/low enhancing sub-regions. Curves overlayed onto histograms are kernel density smoothed estimates from the corresponding sub-regions, and images are shown for the central four of 21 slices

Image volumes were resampled using bilinear interpolation to give 1 × 1 × 5mm voxels, after which multi-slice segmentations were drawn by a clinical fellow (SS) to cover the whole tumour and checked/corrected by a consultant radiologist with 7 years’ experience (DAD). Two different image sub-segmentation methods were explored: sub-segmentation into visually apparent high and low enhancing sub-regions, and erosion of the tumour masks by 10mm to generate core and rim masks, see Fig. 2 and supplementary information section S3. This core/rim sub-segmentation matches the spatial analysis approach previously applied to the histopathology data from these patients [2, 3], where it was discovered that more aggressive clones tended to be detected in the outer 10 mm of the tumour sections. As shown in Fig. 2a, the rim and core regions have a bias towards high enhancement and low enhancement respectively, so a more direct sub-segmentation into visually apparent high- and low-enhancing regions may generate radiomic signatures that are easier to interpret. An algorithm for automatically sub-segmenting into high- and low-enhancing regions was developed, see supplementary information section S4. Three ROI sets were therefore evaluated by generating radiomics features from each sub-segmentation: 1) whole tumour, 2) whole tumour plus high- and low-enhancing sub-regions, 3) whole tumour plus core and rim sub-regions.

One hundred five radiomics features were computed in compliance with IBSI guidelines using pyradiomics v3.0.1 [13, 14], and included 14 shape, 18 first-order and 73 texture features (2D). See supplementary information section S5 for details of extraction parameters and the specific features computed within these three feature groups.

### Feature selection methods

#### Feature reproducibility

Ten randomly selected patients were independently segmented by a second radiologist (JS) and the intra-class correlation coefficient (ICC) was used to reject non-reproducible features with ICC < 0.75. From the 105 computed features, this resulted in the following feature counts for each ROI set: whole tumour = 73, low-enhancing = 48, high-enhancing = 56, tumour core = 72, tumour rim = 58.

#### Unsupervised correlation-based feature reduction

Two correlation-based feature reduction (CFR) methods were evaluated. They both work by iteratively discarding one feature from the most correlated pair of features, but they differ in how they choose which feature to discard during each iteration.

Firstly, the standard CFR method computes the mean absolute correlation between both features and the other remaining features, and the feature with the highest mean correlation is removed. An example of this algorithm is implemented by the function *findCorrelation* from the *caret* toolbox in R and is widely used in radiomics studies.

A limitation of this approach is that ignoring information on feature interpretability could result in a radiomics model that includes features that are harder to interpret. We therefore propose a hierarchical CFR approach, where a hierarchy of feature groups is pre-specified based on an overall judgment of the interpretability of the features within each group. On comparison of two highly correlated features the feature in the lower-ranking group will be discarded. When two features are in the same feature group, the standard CFR rule is applied to determine which feature to discard. The following hierarchy is used here: *MeshVolume* > *shape* > *first-order* > *texture*. Therefore, if a shape feature is highly correlated with a first-order or texture feature, the shape feature is always retained, or if a first-order feature is highly correlated with a texture feature then the first-order feature will be retained. Since tumour volume (i.e. *MeshVolume*) has a straightforward interpretation and is often informative, it is placed in its own group and will always be retained.

#### Supervised feature group selection

**Fig. 3:**
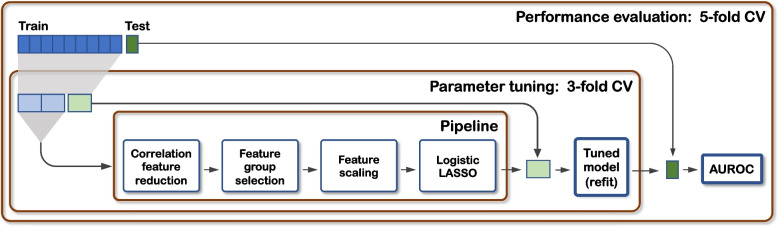
Flow diagram showing the model discovery pipeline within nested cross-validation loops (CV) to perform parameter tuning and performance evaluation

Echoing the hierarchical CFR concept, an additional feature selection step is proposed that restricts the features used for model building to a set of pre-defined feature groups:

1. MeshVolume
2. shape features (including MeshVolume)
3. first-order features
4. texture features

and﻿ this set is augmented with all pair-wise combinations of groups 2–4 plus a final group that includes all features, giving eight feature groups in total. The CFR and feature group selection steps are applied prior, and in addition to any other feature selection steps that may be part of the classification model fitting, e.g., the classifier used in this work includes LASSO regularization that employs embedded feature selection [15]. To avoid under- or over-fitting, selection of the feature group is treated as a tuning parameter of the overall pipeline and is determined using cross-validation (CV), see Fig. 3.

### Classification pipeline and performance evaluation

The feature group selection step can be used in combination with any classification model, but as a proof-of-concept, Logistic regression (LR) with LASSO regularisation was used here since the non-zero LR model coefficients can be used directly for model interpretation (they are equivalent to variable importance estimates [16, 17] that require additional post-processing steps for many other classification models, such as SVM and RF). As required by the LR-LASSO model, radiomics features were standardized using z-scores prior to model fitting.

Two pipelines were evaluated: Conventional, using standard CFR with LR-LASSO and no feature group selection, and Proposed, using hierarchical CFR with feature group selection and LR-LASSO. In each case parameter tuning of the feature group selection and/or the LASSO regularisation coefficient was performed using a grid search with 3-fold CV, nested inside an outer 5-fold CV (repeated 100 times with different random seeds) for performance evaluation, see Fig. 3 and supplementary information section S6. The area under the receiver operating curve (AUROC) was used as a performance metric (averaged across the CV splits), and permutation testing of the entire nested CV procedure was used to compute a *p*-value on the null hypothesis: AUROC = 0.5 [18]. A further model fit was applied to the whole data set for each target (i.e., dispensing with the outer CV but including the inner tuning CV) in order to generate a single model to be used for model interpretation, and this is referred to as the ‘final model’.

The ML pipelines and validation routines were implemented using the Python sklearn toolbox, and both CFR algorithms and the group feature selection step were implemented by extending the sklearn.base.BaseEstimator class.

### Model interpretation

For the Proposed pipeline, the selected feature group can be used to guide model interpretation, whereas for the Conventional pipeline further post-processing of the features with non-zero LR coefficients is needed to determine which feature groups are present in the final model (see Table 2). This was applied to the final models for each target and pipeline and to each of the 500 models obtained from the outer CV splits. The frequency that each feature group was selected across the 500 CV splits was determined to indicate uncertainty and model robustness.

**Table 2:** Classification results for 11 targets of interest that have *p* < 0.05 for one or more ROI sets. For both pipelines the cross-validated AUROC and permutation *p*-values are shown. The Feature group column for the Conventional pipeline details which feature groups are present in the features from the fit to the whole data set. The Feature group column with the Proposed pipeline is the feature group that was selected in the group selection step of the pipeline. The number of features in the final model for each case are shown, and the row in bold for each target is the sub-segmentation that has the highest AUROC for the Proposed pipeline. *EvoST*  = Evolutionary subtype

**Target**	**ROI set**	**Conventional pipeline**				**Proposed pipeline**			
		**AUROC**	***p*-value**	**Feature group**	**# features**	**AUROC**	***p*-value**	**Feature group**	**# features**
Necrosis	whole	0.841	< 0.01	all	9	0.817	< 0.01	all	9
	whole/high/low	0.827	< 0.01	all	11	0.815	< 0.01	shape	7
	whole/rim/core	0.856	< 0.01	all	11	**0.834**	**< 0.01**	**all**	**10**
Sarcomatoid change	whole	0.654	0.17	all	9	0.642	0.13	shape|firstorder	4
	whole/high/low	0.757	0.044	all	8	**0.764**	**0.026**	**shape**	**7**
	whole/rim/core	0.694	0.077	all	7	0.692	0.076	shape|firstorder	2
Microvascular invasion	whole	0.813	< 0.01	all	7	**0.783**	**0.010**	**all**	**7**
	whole/high/low	0.791	< 0.01	all	10	0.750	0.011	all	11
	whole/rim/core	0.812	< 0.01	all	8	0.774	0.010	all	11
Renal vein invasion	whole	0.862	< 0.01	all	8	**0.862**	**< 0.01**	**shape**	**3**
	whole/high/low	0.845	< 0.01	all	7	0.841	< 0.01	shape	8
	whole/rim/core	0.848	< 0.01	all	10	0.852	< 0.01	shape	3
Inferior vena cava invasion	whole	0.705	0.035	all	5	**0.680**	**0.048**	**shape**	**2**
	whole/high/low	0.691	0.033	all	8	0.658	0.064	shape	4
	whole/rim/core	0.730	0.040	all	7	0.672	0.092	shape	3
Overall stage 12 vs 34	whole	0.915	< 0.01	all	9	**0.901**	**< 0.01**	**all**	**10**
	whole/high/low	0.890	< 0.01	all	15	0.893	< 0.01	shape	7
	whole/rim/core	0.904	< 0.01	all	12	0.892	< 0.01	all	12
Branched EvoST	whole	0.720	0.014	all	11	**0.736**	**0.012**	**firstorder**	**2**
	whole/high/low	0.706	0.032	all	10	0.698	0.032	texture	10
	whole/rim/core	0.701	0.044	all	7	0.699	0.035	firstorder	4
Linear EvoST	whole	0.655	0.14	all	8	0.809	0.030	MeshVolume	1
	whole/high/low	0.673	0.19	all	3	**0.822**	**0.031**	**MeshVolume**	**1**
	whole/rim/core	0.632	0.19	all	5	0.814	0.015	MeshVolume	1
ITH Index	whole	0.734	0.012	all	6	**0.745**	**0.013**	**shape**	**2**
	whole/high/low	0.755	0.022	all	14	0.728	0.015	all	13
	whole/rim/core	0.729	0.015	all	8	0.730	0.014	shape|firstorder	4
wGII Max	whole	0.718	0.012	all	3	**0.737**	**0.015**	**shape**	**2**
	whole/high/low	0.696	0.018	all	3	0.704	0.019	shape	5
	whole/rim/core	0.677	0.019	all	3	0.721	0.014	shape	2
Loss 9p21.3	whole	0.793	< 0.01	all	5	**0.814**	**< 0.01**	**shape**	**2**
	whole/high/low	0.779	< 0.01	all	9	0.788	< 0.01	shape	5
	whole/rim/core	0.764	0.010	all	7	0.787	0.011	shape	2

### Experimental evaluations

Classifiers for all 20 targets were built using the model and validation procedures outlined above for both pipelines and for all three ROI sets. Classification performance was evaluated using the AUROC and permutation *p*-values, and model interpretation was performed as outlined above.

## Results

### Model performance assessment

**Fig. 4:**
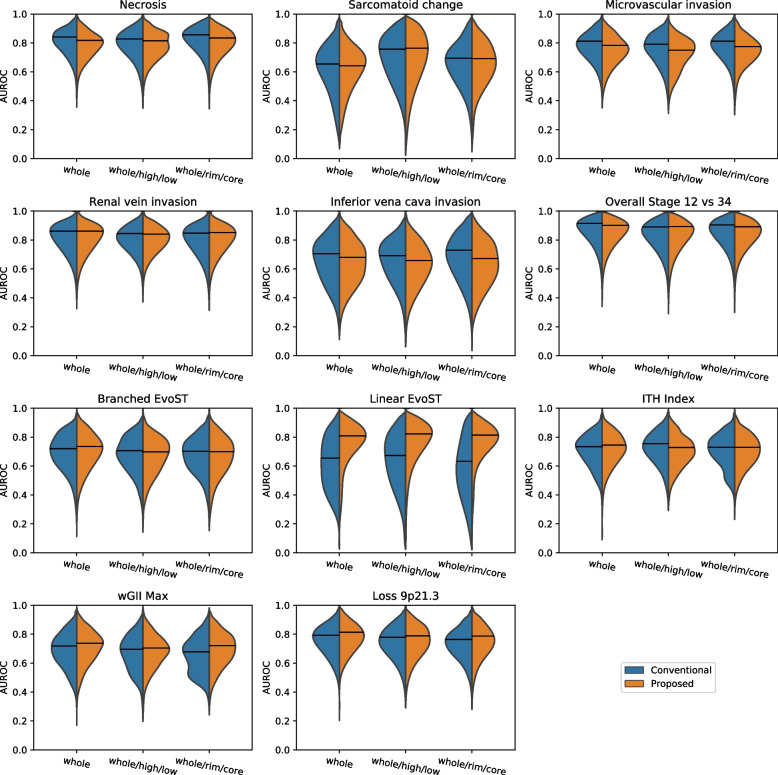
Violin plots showing the distribution in AUROCs across repeated cross-validation obtained for different targets with different pipelines (Conventional and Proposed) and sub-segmentation feature sets (whole, whole/high/low, whole/core/rim). The horizontal lines are the mean AUROCs and in almost all cases the difference in the mean value between the Conventional and Proposed pipelines is small compared to the variation across the cross-validation splits

Figure 4 shows violin plots of the distribution of AUROCs obtained under repeated CV for 11 of the 20 targets. These **targets of interest** had permutation *p*-values that were significant (*p* < 0.05) for at least one ROI set, see Table 2. (For completeness, Table S3 (supplementary information) gives summary performance metrics for 9 targets that had non-significant *p*-values (*p* > 0.05).) Figure 4 shows that despite differences in the feature selection methodologies of the two pipelines, the AUROC performance is within ± 0.05, which is smaller than the variation in AUROC across the CV splits. An exception is for Linear EvoST, where the Proposed pipeline performance is notably higher. Thus, our new methodology does not compromise performance, and in the following sections we demonstrate its impact on interpretability.

### Feature group selection

The Feature group columns in Table 2 show that the Conventional pipeline resulted in final models containing at least one feature from every feature group in all 33 cases, but in only 8/33 cases for the Proposed pipeline. The number of features in the final model was smaller for the Proposed pipeline in 24/33 cases, and for renal vein invasion (RVI), inferior vena cava invasion (IVC), wGII Max and Loss 9p21.3 the final model only involved shape features. We suggest that the more parsimonious models discovered by the Proposed pipeline are preferrable since they avoid over-interpreting or over-fitting the data.

**Fig. 5:**
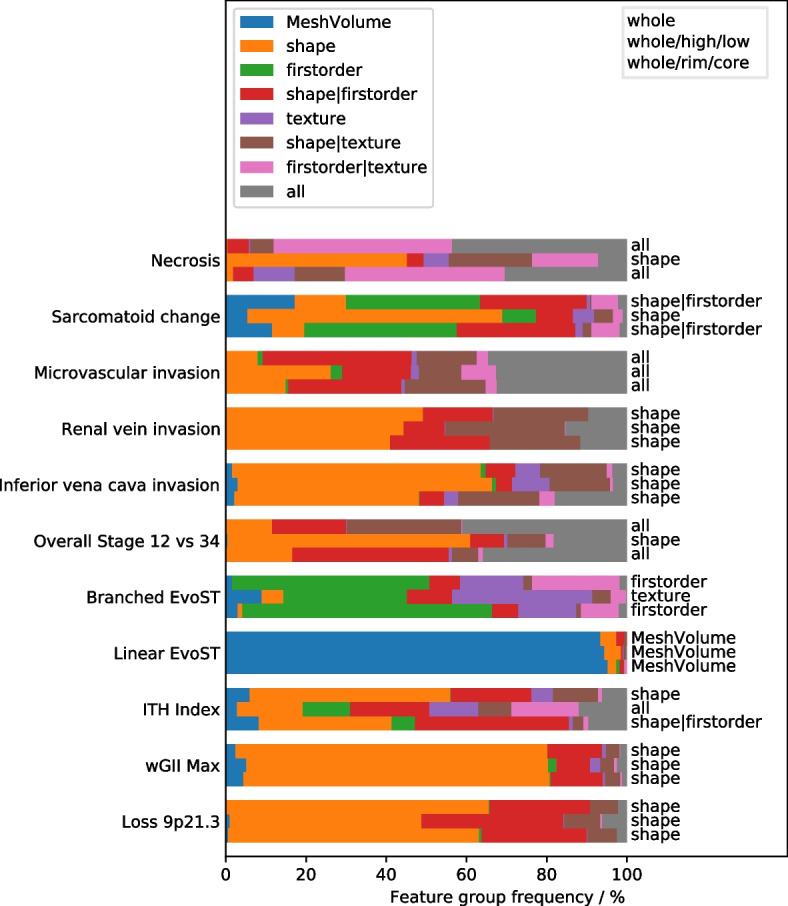
Analysis of which feature groups are selected for the targets of interest when feature group selection is used. The coloured bars show the frequency that each feature group was selected for each target across the repeated CV splits. For each target three sub-segmentation feature sets were used (indicated by upper right box) and the text on the right-hand side indicates which feature group was selected when a single model was estimated from the whole data set – in most cases this corresponds to the feature group with the widest coloured bar. EvoST = evolutionary subtype

Figure 5 presents a robustness analysis of the feature group selection step for the Proposed pipeline. Selecting the same feature group with high frequency in the CV splits is indicative of a stable model that is more likely to generalise well for new data.

With the Proposed pipeline, the shape feature group was selected in > 50% of CV splits for one or more ROI sets for Sarcomatoid change, RVI, IVC invasion, Overall Stage, wGII Max, and Loss 9p21.3, while MeshVolume was selected for Linear EvoST in > 90% of CV splits. For Sarcomatoid change and Overall Stage, the shape feature group was selected in > 60% of CV splits, buts only for models built using the whole/high/low ROI set.

Table 2 shows that features from all feature groups were present in the final model in all 33 cases with the Conventional pipeline, and further analysis demonstrated that this was true for all 500 CV splits in every case. Stability analysis of this kind is therefore not feasible for the Conventional pipeline and is only made possible by applying hierarchical CFR and the feature group selection step, as in the Proposed pipeline.

### Model interpretations for specific targets

Here we interpret the results from the Proposed pipeline in more detail for the eleven targets of interest, grouped according to targets with similar observed patterns in the features selected in their respective models.

#### Necrosis, sarcomatoid change, overall stage 12 vs 34

These three targets are linked by the finding that models built using the whole/high/low ROI set involve only shape features (Table 2), whereas the other two ROI set models also include first-order and/or texture features, making interpretation more challenging for these ROI sets. For necrosis and overall stage, the whole/high/low models involved fewer features than the other ROI sets, while for sarcomatoid change, only the whole/high/low model reached significance (*p* = 0.026). Figure 5 shows that whole/high/low models involving only shape features were selected in > 60% of CV splits for sarcomatoid change and overall stage, and in > 45% of CV splits for necrosis, suggesting that this finding is relatively stable. The models for these targets obtained from the whole/high/low ROI set are therefore of interest for further study in the larger cohort of 320 patients.

Overall stage is a measure of tumour size and spread to other organs and lymph nodes. Our results indicate that higher stages are associated with the presence of necrosis: the necrosis status concurs with the overall stage for 71/91 tumours. Furthermore, all tumours with sarcomatoid change had both necrosis and overall stage 3 or 4. Such associations between the target variables may explain why models from the whole/high/low ROI set use only shape features for all three targets.

Figure 2 shows the high and low enhancing sub-regions are clearly visible in this tumour, which is typical for most cases in this study. Areas of macroscopic necrosis are expected to be contained within the low enhancing sub-regions (although the cause of low enhancement is not limited to necrosis), which may explain why sub-segmentation into high and low enhancing regions is useful for predicting these targets. However, it seems a priori plausible that first-order or texture features (rather than shape features) from these sub-regions may also be predictive on the basis that these features could be sensitive to the presence of necrosis in the low-enhancing sub-region. Two hypotheses warrant further investigation in the final cohort of 320 patients. 1) Only shape features of the sub-regions are informative, so developing shape metrics with increased sensitivity may improve performance. 2) The signal from first-order or texture features is also informative and would boost performance but is too small to be detected in a sample of 91 tumours.

#### RVI, IVC, ITH index, wGII Max, Loss9p21.3

The AUROC performance in these targets is between 0.680 and 0.862, and the common theme is that all five targets are associated with the shape feature group for the whole ROI set (Table 2 and Fig. 5). In all five targets the highest AUROC is achieved with the whole ROI set, suggesting that the shape of the tumour itself is predictive – image heterogeneity (via texture and first-order features) within the tumour is not informative, and sensitivity to image heterogeneity is not enhanced by either sub-segmentation method.

The features and LR coefficients of the best final models are detailed in Table 3, and for all targets the positive class is associated with lower Sphericity values (indicating more flattened tumour shapes) and larger tumour volumes. Sphericity is a dimensionless feature defined as $\sqrt[3]{36\pi {V}^{2}}/A$, ($V$=volume, $A$=surface area), and it reaches a maximum value of 1 for a sphere and 0 for a disk. Flatness (present in the RVI model only) is defined via the principal components of the tumour shape [13] and takes values in the same range. Whilst Sphericity and Flatness are sensitive to similar aspects of the tumour shape, the Spearman correlation between them in these data is 0.58, indicating they contain somewhat independent information.

**Table 3:** Logistic regression coefficients, AUROC and *p*-values for five targets that are robustly predicted by three shape features from the whole tumour ROI using the Proposed pipeline

**Target**	**Logistic regression coefficients**			**Performance**	
	**Sphericity**	**Flatness**	**MeshVolume**	**AUROC**	***p*-value**
Renal vein invasion	-1.278	-0.551	0.246	0.862	< 0.01
IVC invasion	-0.830		0.094	0.680	0.048
ITH Index	-0.722		0.528	0.745	0.013
wGII Max	-0.783		0.230	0.737	0.015
Loss 9p21.3	-1.29		0.367	0.814	< 0.01

The associations between shape features and ITH index, wGII Max and Loss9p21.3 are consistent with previous findings showing that these targets are associated with tumour growth modes, such as surface or volume growth [19]. These radiomic findings suggest that developing and evaluating more advanced shape metrics may yield improved classification performance and insights into these relationships.

RVI and IVC invasion are processes that are sometimes detectable on CT scans by inspection of the relevant vascular structures, but these image regions are not included in the ROIs used here for radiomic analysis. However, an association between these targets and tumour volume is plausible since larger tumours are more likely to impinge on surrounding structures. Follow-up work in the full cohort of 320 patients is needed to determine if these associations are reproducible, and a case-by-case analysis is warranted to further elucidate this finding.

#### Linear EvoST

The Proposed pipeline results indicate that MeshVolume is associated with Linear EvoST in a univariate model. For all three ROI sets, the Proposed pipeline selected only the MeshVolume of the whole tumour, and the LR coefficients were negative, indicating an inverse association. Linear EvoST tumours are known to be smaller than branched or punctuated EvoSTs suggesting they may represent an earlier stage of the evolutionary trajectory [3]. The Conventional pipeline was not able to uncover this association in models built using any of the ROI sets.

#### Branched EvoST

The highest performing model for this target used the whole ROI set and contained two first-order features: uniformity and mean. The LR coefficient signs indicate that the Branched EvoST is associated with higher image heterogeneity (i.e., lower uniformity values) and higher mean HU values. The first-order feature group was selected in 49% of CV splits, indicating this finding is relatively stable, but more data are needed to discover if this result is generalisable.

#### Microvascular invasion (MVI)

MVI can be predicted with good performance (AUROC > 0.75) and is the only feature where every ROI set resulted in models that contain features from all feature groups. Figure 5 shows that there is also considerable variability in which feature group is selected, suggesting that this target would benefit from more data to support any detailed interpretation.

For all targets except MVI, the models obtained using the Proposed pipeline that are most relevant for follow-up study involve only shape features or first-order features, and so MVI is the only target where texture features appear to be informative. MVI is a histopathological finding related to both vessel co-option (invasion of existing vessels) and angiogenesis and is a process occurring at the capillary length-scale, i.e., somewhat smaller than the CT voxel size, and so spatial variability in the structure of the vascular invasion processes may give rise to variations in pixel intensity that appear as altered image texture. Further study in the larger cohort is needed to further explore this hypothesis.

Since the models discovered by the Proposed pipeline for this target involve features from all feature groups, this target benefits the least from using the Proposed pipeline. This is evidenced by the AUROC increase, smaller *p*-value and lower number of features for all ROI sets with the Conventional pipeline compared to the Proposed pipeline for MVI prediction. Consideration of the impact of the feature group selection step suggests that this finding is to be expected since for every CV split where the ‘all’ feature group is not selected, the LR model will not have access to some of the features that would lead to good performance, and so the AUROC value for that CV split will be biased downward. The Conventional pipeline essentially selects the ‘all’ feature group for every CV split, and so is not subject to this effect.

## Discussion

We have demonstrated that 11 of 20 targets of interest can be predicted with an AUROC significantly greater than 0.5 (*p* < 0.05), including six histopathological, two EvoST, ITH index (intratumoral heterogeneity), wGII Max (chromosomal complexity) and one somatic alteration marker (Loss 9p21.3). Putative interpretations of the models generated from the Proposed pipeline have been presented for these 11 targets, of which five can be predicted with an AUROC > 0.8 (Necrosis, Renal vein invasion, Overall stage, Linear EvoST, Loss 9p21.3), and are therefore targets of interest for further validation in the final TRACERx Renal cohort of 320 patients. This would represent a significant step forward in clinical practice by permitting improved patient risk stratification using non-invasive investigations.

Contrary to previous studies [20–22], we were unable to predict BAP1 or PBRM1 status. However, sample sizes in previous studies were small (the largest comprising only 65 patients) and sampling bias arising from genetic profiling of a single tumour sample region can cause increased false negative detection rate. In TRACERx Renal, multi-region sampling permits more sensitive detection of somatic events determined by the presence of the alteration in any of the tumour regions sampled (one sample per cm^2^ of the tumour slice).

Radiomic predictions of the clonal status of the somatic alterations were also explored, but none of these targets could be predicted in this study. It is likely that radiological changes associated with different clones of the same genetic locus will be more subtle than changes associated with different genetic loci, and thus it is reasonable to assume that detecting mutational clonality from radiological imaging will be particularly challenging.

Prediction of the punctuated EvoST did not reach significance in these data which may be due to the one vs all classification that was used for the EvoSTs due to the small sample size in this study. Multinomial classification models may be required to handle the EvoST using radiomics, and the complete cohort of 320 patients will provide a more suitable data set for such development.

Two pipelines have been compared: the Conventional pipeline reflects standard practice in radiomics studies, and the Proposed pipeline includes two feature selection innovations tailored to radiomics studies that are designed to improve model interpretability. The AUROC performance is very similar for the Conventional and Proposed pipelines, suggesting that there is a high degree of redundancy between the radiomics features such that multiple combinations of features result in models with similar performance. This is a well-known effect with high-dimensional feature sets when the amount of training data is limited.

Generic feature selection techniques, whether they are based on filter, wrapper, or embedded methods, only consider the predictive potential of each feature, and not their interpretability. In the context of feature sets that exhibit redundancy, there is therefore no guarantee that the use of generic feature selection techniques will result in a model that has the most straightforward interpretation. The results for models predicting RVI, IVC, ITH index, wGII Max and Loss9p21.3 are a clear demonstration of this – the AUROC estimates for the Conventional and Proposed pipelines are equivalent, but the Proposed pipeline selected fewer features from only the shape feature group for these targets. The LR coefficient values from the Conventional pipeline are given in Table S2 for these targets showing that whilst Sphericity is the top feature in each case, first-order and texture features appear with non-negligible coefficient magnitudes, and MeshVolume is notably absent from all instances leading to quite different interpretations to the Proposed pipeline models.

The hierarchical CFR is a key innovation in the Proposed pipeline and arises from the idea that a judgement on the a priori interpretability of features from different feature groups can be used to intentionally bias the correlation-based feature selection step. Four feature groups were used in this study, but additional feature groups may be necessary with different radiomics feature sets. For example, filtered image features (wavelets, Laplacian of Gaussian, etc.) involve additional processing steps and may be placed after texture features in the hierarchy, whereas previously validated semantic imaging features (e.g., PIRADS features) may be placed before all the radiomics feature groups. In general, the availability of a meaningful interpretation and/or biological or clinical validation of the features within each group should be used to determine the group hierarchy.

It is interesting to note that the use of hierarchical CFR is critical for this data set because the MeshVolume and glszm_GrayLevelNonUniformity (glszm_GLNU) features from the whole tumour ROI have a Spearman correlation of 0.979. In this case, application of the standard CFR algorithm to these data results in MeshVolume being rejected in favour of glszm_GLNU. In those targets where MeshVolume is an important predictor, use of the standard CFR algorithm would mean the feature group selection step would be forced to retain the texture feature group, which would confound interpretation. This has clearly impacted the Conventional pipeline models for ITH index, wGII Max and Loss9p21.3 (see Table S2) where additional texture features are present in these models compared to those for the same targets in Table 3.

Tumour volume is a sufficiently well-known predictor that specific checks of the predictive power of this feature are included in some published radiomics studies. This is reflected in the hierarchy used here – MeshVolume is given special status by placing it in the first feature group, so it is always retained if it is correlated to any other feature. Texture features are placed at the other end of the hierarchy on the basis that they are in general more difficult to interpret – in many papers where texture features are discovered to be predictive, the interpretation given is that they are associated with “tumour heterogeneity”. Whilst this may be true, to fully elucidate the correlations between texture features and biological processes in the spirit of Tomaszewski and Gilles [7], more specific biological interpretations of texture features are needed. Developing such interpretations is not the intention of this work, but rather we aim to develop a methodology that only retains texture features if there is sufficient evidence to do so. Therefore, when a pipeline does select a texture feature it will be more likely that it is genuinely contributing texture information to the model, and so any attempt to link this to more well-understood biological correlates will have a greater chance of success.

Combining the hierarchical CFR with an explicit group selection step is designed to increase the ability of the Proposed pipeline to reject features that are not informative. Treating the group selection as a tuning parameter which is optimised using an inner CV has two potential benefits. Firstly, the frequency with which each feature group is selected across the CV splits can be measured, which gives information on model robustness. Secondly, the use of CV for parameter tuning allows the complexity of the model (i.e., how many feature groups are selected) to adapt to the data, both in terms of any informative patterns in the data, but also the data set size. Feature groups that lead to consistent performance improvements across the CV splits are likely to be selected, and this stability will tend to be greater in larger data sets since the different data splits will be statistically more similar. Conversely, if a feature group contains some weakly predictive features, then for a small data set the feature group is likely to be selected in fewer CV splits, and this can be detected in the feature group selection frequency.

Sub-segmentations were used in this study for two reasons. Firstly, spatial analysis of the surgical specimens in the TRACERx study demonstrated different genomic profiles in tissue samples taken from the core and rim of the tumour [23]. We therefore hypothesized that if these differences also affect the CT images, then an equivalent sub-segmentation may yield a richer radiomics feature set with greater predictive potential. Figure 2 presents a typical example patient showing that whilst a geometric definition of the core and rim captures some of the image variation, an image-led sub-segmentation into high and low enhancing sub-regions may capture the heterogeneity present in the CT images more accurately. The low enhancing sub-region is associated with, but not specific to necrosis, and so it was hypothesized that prediction of necrosis may be feasible using sub-segmentation. The results in Table 2 and Fig. 5 indicate that whilst necrosis can be predicted using CT radiomics, more data is needed to show which ROI set is most predictive, both in terms of prediction performance, and in the stability of the feature group selection. However, for sarcomatoid change and overall stage, Fig. 5 shows that there is strong evidence that the whole/high/low ROI set leads to models with simpler more interpretable features, and also improved performance for predicting sarcomatoid changes.

An advantage of the proposed group selection method is that it can be applied in conjunction with any predictive model (e.g. support vector machines, random forest etc.), which is in contrast to the priority LASSO [24, 25] and the group LASSO [26] which have a similar aim, but can only be used with models that include LASSO regularisation. The proposed approach is more general than the priority LASSO as it considers all feature group combinations, whereas the priority LASSO considers a restricted set of combinations obtained by adding groups in a stepwise manner according to some pre-determined priority sequence. Compared with the group LASSO, the proposed method differs in the form of the regularisation used, and consequently the level of sparsity and interpretability of the final models. The group LASSO uses L_1_ regularisation between groups and L_2_ regularisation within groups, whereas the proposed method uses L_0_ between groups and L_1_ within groups. L_0_ and L_1_ regularisation both result in sparse solutions, where unimportant features or feature groups are removed from the model, whereas with L_2_ regularisation the coefficients for unimportant features are shrunk towards zero, but they are still present in the model.

An important limitation of this study is the small sample size, which was also a key driver for the development of the proposed feature selection methods. On completion, the TRACERx study aims to recruit 320 patients, and so further work applying the proposed methods in this larger cohort will be of interest. A consequence of the small sample size is that the radiomics feature sets analysed here did not include the extended features available in pyradiomics that are derived using image filtering (e.g. wavelet filtering) as their inclusion would risk over-fitting. Future work investigating the impact of the feature group selection methods with these extended feature sets in a suitable data set is of interest.

## Conclusions

In conclusion, we have presented two novel feature selection strategies for use with radiomics studies and demonstrated that their combined use yields models with equivalent performance to a widely used pipeline. The proposed strategies also lead to models with fewer features meaning model interpretation is more straightforward across a range of eleven molecular, histopathological and clinical targets. In addition, use of image sub-segmentations has been assessed, and preliminary analysis found improvements to classification model performance and interpretability for a subset of the targets.

### Supplementary Information

**Additional file 1: S1.** Patient selection, inclusion and exclusion criteria **S2.** Evolutionary subtype definition and histopathological specimen preparation. **﻿Figure S1.** Summary of key conclusions of the TRACERx Renal study [2, 3]. **S3. **Tumour segmentation. **S4.** High/low enhancing sub-segmentation algorithm. **S5.** Radiomics feature extraction. **S6.** Parameter tuning and performance evaluation. **Table S1.** Details of scanner settings separated on four scanner vendors. **Table S2.** Features and LR coefficients for models derived using the Conventional pipeline from the whole ROI set for the five targets. **Table S3.** Classification results for 9 targets that have non-significant *p*-values (>0.05) for all models. 

## Data Availability

The summarised molecular data used in this work is published in supplementary form in [2, 3] and the corresponding raw data is available on request via the same source. The radiomics data (table of feature values) for the subjects in this study are available on request from the corresponding author, and we plan to make the DICOM image data available when the TRACERx renal trial is complete.

## Abbreviations

AUROC: Area under receiver operating characteristic
ccRCC: Clear cell renal cell carcinoma
CE-CT: Contrast-enhanced CT
CFR: Correlation-based feature reduction
CV: Cross-validation
EvoST: Evolutionary subtype
glszm_GLNU: glszm_GrayLevelNonUniformity
ICC: Intra-class correlation coefficient
ITH: Intratumoural heterogeneity
IVC: Inferior vena cava invasion
LASSO: Least absolute shrinkage and selection operator
LR: Logistic regression
ML: Machine learning
NGS: Next generation sequencing
PACS: Picture archiving and communications system
ROI: Region of interest
RVI: Renal vein invasion
SCNAs: Somatic copy number alterations
wGII: Weighted genomic instability index
XNAT: Extensible neuroimaging archive toolkit
