# Supplementary material for: Interpretability of radiomics models is improved when using feature group selection strategies for predicting molecular and clinical targets in clear-cell renal cell carcinoma: insights from the TRACERx Renal study
Source: Cancer Imaging. 2023 Aug 14;23:76. doi: 10.1186/s40644-023-00594-3 (PMC10424427; doi:10.1186/s40644-023-00594-3)
Supplement: Supplementary file 1 — Additional file 1: S1. Patient selection, inclusion and exclusion criteria S2. Evolutionary subtype definition and histopathological specimen preparation. ﻿Figure S1. Summary of key conclusions of the TRACERx Renal study [2, 3]. S3. Tumour segmentation. S4. High/low enhancing sub-segmentation algorithm. S5. Radiomics feature extraction. S6. Parameter tuning and performance evaluation. Table S1. Details of scanner settings separated on four scanner vendors. Table S2. Features and LR coefficients for models derived using the Conventional pipeline from the whole ROI set for the five targets. Table S3. Classification results for 9 targets that have non-significant p-values (>0.05) for all models. [file 40644_2023_594_MOESM1_ESM.docx]

## Supplementary Information

### **S1 Patient selection, inclusion/exclusion criteria**

Exclusions were due to incorrect contrast phase (7 cases), axial images unavailable (4 cases), only MR scans available (1 case), imaging artefact over tumour (1 case), missing/corrupted imaging data (2 cases): total 15 exclusions with imaging available for 86 patients. Three patients had bilateral tumours, two patients had two sites of disease in the same kidney giving a final total of 91 lesions for radiomics analysis.

### **S2 Evolutionary subtype definition and histopathological specimen preparation**

Clinical presentations of ccRCC are diverse, ranging from indolent small renal masses to large aggressive tumours. Patterns of metastases are varied, being rapid and widespread, delayed and oligometastatic or latent and single (Fig. S1). Following surgical resection one third of patients with localised disease relapse, but accurate risk stratification is challenging.

Predicting clinical trajectory is fundamental to improving patient care made possible by understanding the evolutionary course of the primary tumour (Turajlic, S., et al., Cell, 2018. 173(3): p. 595-610). Tumour evolution can be defined by highly conserved patterns of ordering, co-occurrence and mutual exclusivity of genomic events. In ccRCC seven evolutionary subtypes are grouped into three categories, or “modes” of evolution.

The three distinct genetic evolutionary modes are characterised by differing levels of genetic intratumoral heterogeneity (ITH), chromosomal complexity (expressed as weighted genomic instability index, wGII), and are associated with clinical outcomes (Fig. S1). Specifications are below, and more details are in (Turajlic, S., et al., Cell, 2018. 173(3): p. 581-594 and p. 595-610).

**Punctuated evolution**: characterised by early fixation of a highly fit clone resulting in low ITH, high wGII and associates with rapid primary tumour growth with widespread metastases; these tumours do not benefit from cytoreductive nephrectomy or metastasectomy.

**Branched evolution**: characterised by gradual accumulation of genetically distinct subpopulations of cells resulting in high ITH and high wGII and associates with slow primary tumour growth, and solitary/oligometastases; these tumours benefit from surgery in all settings.

**Linear evolution**: Tumours have low ITH and low wGII and limited potential for progression suggesting they could be managed by surveillance alone.


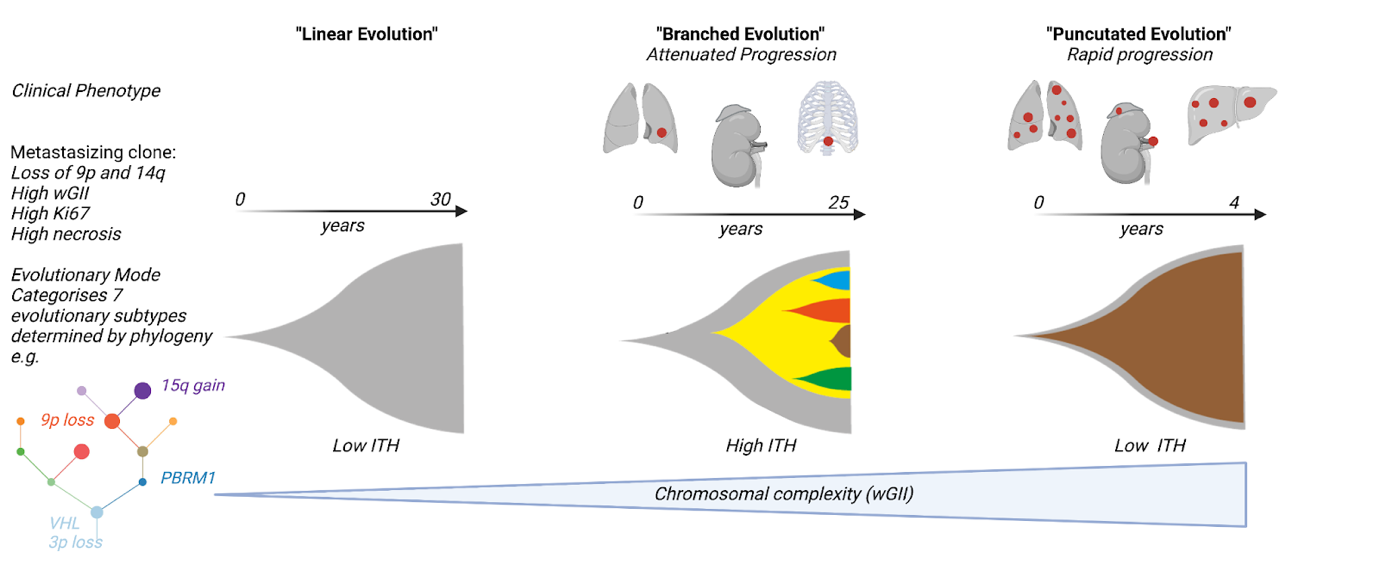


**Figure S1.** Summary of key conclusions of the TRACERx Renal study(Turajlic, S., et al., Cell, 2018. 173(3): p. 581-594 and p. 595-610). Three modes of evolution underpinned by differing levels of ITH and chromosomal complexity, (wGII), associate with distinct clinical behaviour.  The modes of evolution are further defined by conserved ordering, co-occurrence and mutual exclusivity of mutations in driver genes, including *VHL*, *PBRM1*, *SETD2* and *BAP1*. Metastatic competence is afforded by high levels of SCNAs and loss of *9p* and *14q*.

### **S3 Tumour segmentation**

Multi-slice segmentations were drawn by a clinical fellow (SS) to cover the whole tumour and checked by a consultant radiologist with 13 years’ experience (DAD). In cases that contained significant calcifications, separate contours were drawn so that these regions could be excluded from the final mask as the very high signal intensities in these voxels tend to have a disproportionate effect on the computed radiomics feature values.

Two different image sub-segmentation methods were explored: 1) erosion of the tumour masks by 10 pixels (10 mm) to generate core and rim masks, 2) sub-segmentation into visually apparent high and low enhancing masks, see figure 1. In both cases radiomic feature sets for the whole tumour and both sub-regions were computed, and predictive models built using the following three combinations: whole tumour, whole and rim and core, whole and high and low.

The use of a 10 mm rim/core sub-segmentation matches the spatial analysis approach previously applied to the histological data from these patients[2,3], where it was discovered that more aggressive clones tended to be detected in the outer 10 mm of the tumour sections. As shown in figure 1(a), the rim and core regions have a bias towards high enhancement and low enhancement respectively, so a more direct sub-segmentation into visually apparent high- and low-enhancing regions may generate radiomic signatures that are easier to interpret. An algorithm for automatically sub-segmenting into high- and low-enhancing regions was developed using a combination of a two component Bayesian Gaussian mixture model, followed by morphological operations to remove any spurious voxels, and is described below.

### **S4 High/low enhancing sub-segmentation algorithm**

1. Get subset of tumour voxels to estimate low/high enhancing class statistics:
   1. Remove voxels outside range: -50 < HU < 200
   2. Remove brightest/darkest 0.5% of voxels
   3. Select every n^th^ voxel to give 20,000 voxels
2. Fit two-class Bayesian Gaussian mixture model using Bayesian EM algorithm to voxels selected in step 1 and compute class probability for all voxels (i.e., including any that are not used in step 1). Prior distributions are m_1_ ~ N(18, 9), m_2_ ~ N(70, 9) for the class means and s_1_^2^, s_2_^2^ ~ IG(2502, 2.78e-6) for the class variances. The class variance prior gives a distribution on the class standard deviations (s_1_ and s_2)_ that has mean = 12 and 95% CI = [11.8, 12.2].
3. Apply Gaussian image filter with smoothing bandwidth = 0.5 voxel to the class map and threshold this at 0.6 to give a binary map for the high and low enhancing regions.
4. Apply morphological operations to tidy up any remaining spurious regions:
   1. Remove blobs or holes that are less than 9 voxels
   2. Remove regions where >50% of voxels are on the boundary of the region (removes narrow regions that are typically due to imaging artefacts)
   3. Remove any high enhancing regions whose area is less than 2% of the largest subregion.
   4. Use a closing operation with radius 2 pixels to further tidy the sub-region masks.

### **S5 Radiomics feature extraction**

Scans for 73/86 patients had a slice thickness of 5 mm, one was 5.8 mm, and the remainder were between 0.6 and 3 mm. The in-plane pixel spacing was 1 mm or less for all scans, so all 3D volumes were resampled using bilinear interpolation (MATLAB imresize function) to give 1 x 1 x 5 mm voxel dimensions.

Radiomics features were computed in compliance with the IBSI, using pyradiomics v3.0.1 (<https://doi.org/10.1158/0008-5472.CAN-17-0339>), and included 14 shape, 18 first-order and 73 texture features (22 glcm, 16 glrlm, 16 glszm, 14 gldm, 5 ngtdm, see below), and the following settings were used: binWidth = 20 HU, force2D = True and weightingNorm = None. The effect of the last two settings is that the texture feature computations only include in-plane interactions between neighbouring voxels, and not interactions between slices, which is appropriate since the slice spacing is much larger than the voxel spacing (5mm, compared to 1mm).

**Shape features (shape)**

MeshVolume

VoxelVolume

SurfaceArea

SurfaceVolumeRatio

Compactness1

Compactness2

SphericalDisproportion

Sphericity

Maximum3DDiameter

MajorAxisLength

MinorAxisLength

LeastAxisLength

Elongation

Flatness

**First-order features (firstorder)**

10Percentile

90Percentile

Energy

Entropy

InterquartileRange

Kurtosis

Maximum

MeanAbsoluteDeviation

Mean

Median

Minimum

Range

RobustMeanAbsoluteDeviation

RootMeanSquared

Skewness

TotalEnergy

Uniformity

Variance

**Gray level co-occurrence matrix features (texture)**

MaximumProbability

JointAverage

SumSquares

JointEntropy

DifferenceAverage

DifferenceVariance

DifferenceEntropy

SumEntropy

JointEnergy

Contrast

Id

Idn

Idm

Idmn

InverseVariance

Correlation

Autocorrelation

ClusterTendency

ClusterShade

ClusterProminence

Imc1

Imc2

**Gray level run-length matrix features (texture)**

GrayLevelNonUniformity

GrayLevelNonUniformityNormalized

GrayLevelVariance

HighGrayLevelRunEmphasis

LongRunEmphasis

LongRunHighGrayLevelEmphasis

LongRunLowGrayLevelEmphasis

LowGrayLevelRunEmphasis

RunEntropy

RunLengthNonUniformity

RunLengthNonUniformityNormalized

RunPercentage

RunVariance

ShortRunEmphasis

ShortRunHighGrayLevelEmphasis

ShortRunLowGrayLevelEmphasis

**Gray level size-zone matrix features** **(texture)** GrayLevelNonUniformity

GrayLevelNonUniformityNormalized

GrayLevelVariance

HighGrayLevelZoneEmphasis

LargeAreaEmphasis

LargeAreaHighGrayLevelEmphasis

LargeAreaLowGrayLevelEmphasis

LowGrayLevelZoneEmphasis

SizeZoneNonUniformity

SizeZoneNonUniformityNormalized

SmallAreaEmphasis

SmallAreaHighGrayLevelEmphasis

SmallAreaLowGrayLevelEmphasis

ZoneEntropy

ZonePercentage

ZoneVariance

**Gray dependence matrix features (texture)**

DependenceEntropy

DependenceNonUniformity

DependenceNonUniformityNormalized

DependenceVariance

GrayLevelNonUniformity

GrayLevelVariance

HighGrayLevelEmphasis

LargeDependenceEmphasis

LargeDependenceHighGrayLevelEmphasis

LargeDependenceLowGrayLevelEmphasis

LowGrayLevelEmphasis

SmallDependenceEmphasis

SmallDependenceHighGrayLevelEmphasis

SmallDependenceLowGrayLevelEmphasis

**Neighbouring gray tone difference matrix (texture)**

Busyness

Coarseness

Complexity

Contrast

Strength

### **S6 Parameter tuning and performance evaluation**

The pipeline has two elements that contain tuning parameters – the feature group selection and the LASSO coefficient – and these were optimised using 3-fold CV with a grid search, nested inside the outer CV, see figure 2. A grid search was used over all combinations of the four feature groups and 10 logarithmically spaced values for the LASSO coefficient (from 0.01 to 1), giving a total of 40 tuning combinations. The tuning parameter combination with the best average performance in the inner CV test data (light green) was used to refit the model to all the data passed to the inner CV (i.e., the light blue and light green boxes combined), and this tuned model was evaluated in the outer CV test data (bright green).

Five-fold stratified CV was used for performance evaluation, and this was repeated 100 times to average over any random fluctuations in the performance estimates from each split, resulting in 500 models fitted to different subsets of the data.

Permutation testing of the entire nested CV procedure was used to compute a p-value on the null hypothesis that the AUROC was equal to 0.5. Following the recommendations in [18] only one repeat of the outer CV was used for each iteration of the permutation testing routine, and 100 permutation samples were generated.

|  | **Philips** | **GE** | **Siemens** | **Toshiba** |
| --- | --- | --- | --- | --- |
| Number of scans | 27 | 21 | 19 | 19 |
| Peak voltage/kV | 120-120 | 100-120 | 100-130 | 120-120 |
| Tube current/mA | 30-332 | 102-723 | 25-454 | 80-440 |
| Reconstruction diameter/mm | 350-500 | 350-510 | 354-512 | 359-500 |
| Reconstruction kernels | B, C | CHST, DETAIL, SOFT, STANDARD | B20f, B30f, B31f, B31s, I26f\3, I30f\1, I41f\2, T20s | FC01, FC03, FC07, FC08, FC10, FC11, FC13, FC17, FC18 |
| Slice thickness/mm | 0.625-5 | 0.73-5 | 0.6-5 | 3-5.8 |
| Pixel spacing/mm | 0.68-1.0 | 0.68-1.0 | 0.69-1.0 | 0.70-1.0 |

**Table S1:** Details of scanner settings separated on four scanner vendors.

| **Target** | **Features** | **LR coefficients** |
| --- | --- | --- |
| Renal vein invasion | shape_Sphericity | -1.117 |
|  | shape_Flatness | -0.536 |
|  | ngtdm_Strength | -0.314 |
|  | glcm_Idmn | 0.292 |
|  | firstorder_10Percentile | -0.074 |
|  | ngtdm_Complexity | 0.050 |
|  | glszm_ZoneEntropy | 0.027 |
|  | glcm_ClusterProminence | 0.003 |
|  |  |  |
| IVC invasion | shape_Sphericity | -0.679 |
|  | ngtdm_Complexity | 0.225 |
|  | glszm_SmallAreaEmphasis | 0.126 |
|  | glszm_ZoneVariance | 0.075 |
|  | ngtdm_Strength | -0.010 |
|  |  |  |
| ITH Index | shape_Sphericity | -0.603 |
|  | glszm_GrayLevelNonUniformity | 0.251 |
|  | glszm_ZoneEntropy | 0.153 |
|  | glszm_ZoneVariance | 0.063 |
|  | glcm_ClusterProminence | 0.013 |
|  | ngtdm_Strength | -0.009 |
|  |  |  |
| wGII Max | shape_Sphericity | -0.708 |
|  | glszm_ZoneEntropy | 0.034 |
|  | glszm_GrayLevelNonUniformity | 0.021 |
|  |  |  |
| Loss 9p21.3 | shape_Sphericity | -1.127 |
|  | glcm_Imc2 | 0.222 |
|  | glszm_GrayLevelNonUniformity | 0.217 |
|  | firstorder_10Percentile | -0.051 |
|  | glszm_ZoneVariance | 0.008 |

**Table S2:** Features and LR coefficients for models derived using the Conventional pipeline from the whole ROI set for the five targets shown. Coefficients are listed in order of descending coefficient magnitude.

|  |  |  | **Conventional pipeline** | | | |  | **Proposed pipeline** | | | |
| --- | --- | --- | --- | --- | --- | --- | --- | --- | --- | --- | --- |
| **Target** | **ROI set** |  | **AUROC** | **p-value** | **Feature group** | **# features** |  | **AUROC** | **p-value** | **Feature group** | **# features** |
| EvoST Punctuated | whole |  | 0.645 | 0.11 | all | 16 |  | 0.63 | 0.10 | firstorder\|texture | 12 |
|  | whole/high/low |  | 0.642 | 0.079 | all | 32 |  | 0.641 | 0.091 | firstorder\|texture | 24 |
|  | whole/rim/core |  | 0.642 | 0.074 | all | 27 |  | 0.631 | 0.11 | firstorder\|texture | 25 |
|  |  |  |  |  |  |  |  |  |  |  |  |
| wGII Median | whole |  | 0.611 | 0.14 | all | 18 |  | 0.609 | 0.10 | shape\|firstorder | 7 |
|  | whole/high/low | | 0.613 | 0.10 | all | 31 |  | 0.618 | 0.092 | shape | 10 |
|  | whole/rim/core | | 0.609 | 0.15 | all | 24 |  | 0.597 | 0.11 | shape | 6 |
|  |  |  |  |  |  |  |  |  |  |  |  |
| Loss 9p21.3 Clonal=TRUE | whole |  | 0.667 | 0.11 | all | 17 |  | 0.639 | 0.11 | firstorder | 3 |
|  | whole/high/low |  | 0.656 | 0.076 | all | 33 |  | 0.631 | 0.10 | shape\|firstorder | 15 |
|  | whole/rim/core |  | 0.684 | 0.090 | all | 25 |  | 0.641 | 0.10 | shape | 6 |
|  |  |  |  |  |  |  |  |  |  |  |  |
| Loss 14q31.1 | whole |  | 0.584 | 0.20 | all | 15 |  | 0.553 | 0.30 | shape | 4 |
|  | whole/high/low | | 0.573 | 0.25 | all | 20 |  | 0.552 | 0.33 | MeshVolume | 1 |
|  | whole/rim/core | | 0.616 | 0.11 | all | 21 |  | 0.588 | 0.16 | texture | 12 |
|  |  |  |  |  |  |  |  |  |  |  |  |
| Loss 14q31.1 Clonal=TRUE | whole |  | 0.486 | 0.59 | all | 8 |  | 0.516 | 0.48 | firstorder | 3 |
|  | whole/high/low |  | 0.511 | 0.46 | all | 21 |  | 0.523 | 0.3701 | firstorder | 5 |
|  | whole/rim/core |  | 0.461 | 0.75 | all | 19 |  | 0.528 | 0.3507 | firstorder | 6 |
|  |  |  |  |  |  |  |  |  |  |  |  |
| BAP1 | whole |  | 0.519 | 0.47 | all | 1 |  | 0.464 | 0.67 | shape | 2 |
|  | whole/high/low | | 0.447 | 0.62 | all | 1 |  | 0.410 | 0.76 | firstorder | 5 |
|  | whole/rim/core | | 0.483 | 0.62 | all | 1 |  | 0.442 | 0.67 | firstorder | 4 |
|  |  |  |  |  |  |  |  |  |  |  |  |
| BAP1 Clonal=TRUE | whole |  | 0.536 | 0.46 | all | 8 |  | 0.442 | 0.62 | firstorder | 3 |
|  | whole/high/low |  | 0.433 | 0.65 | all | 19 |  | 0.412 | 0.73 | MeshVolume | 0 |
|  | whole/rim/core |  | 0.534 | 0.44 | all | 14 |  | 0.448 | 0.60 | firstorder | 6 |
|  |  |  |  |  |  |  |  |  |  |  |  |
| PBRM1 | whole |  | 0.559 | 0.22 | all | 14 |  | 0.542 | 0.31 | firstorder | 3 |
|  | whole/high/low | | 0.557 | 0.29 | all | 22 |  | 0.541 | 0.32 | firstorder | 6 |
|  | whole/rim/core | | 0.595 | 0.22 | all | 22 |  | 0.563 | 0.24 | firstorder | 5 |
|  |  |  |  |  |  |  |  |  |  |  |  |
| PBRM1 Clonal=TRUE | whole |  | 0.504 | 0.47 | all | 9 |  | 0.459 | 0.67 | shape\|texture | 8 |
|  | whole/high/low |  | 0.471 | 0.67 | all | 19 |  | 0.453 | 0.73 | shape | 3 |
|  | whole/rim/core |  | 0.500 | 0.51 | all | 20 |  | 0.445 | 0.67 | shape\|texture | 15 |

**Table S3:** Classification results for 9 targets that have non-significant p-values (>0.05) for all models. EvoST = evolutionary subtype.
